# Suicidal behaviour and addiction: An inseparable couple? Mechanisms underlying the association and targets for interventions

**DOI:** 10.1192/j.eurpsy.2021.56

**Published:** 2021-08-13

**Authors:** A. Lengvenyte

**Affiliations:** 1 Department Of Emergency Psychiatry And Acute Care, Psnrec, Univ Montpellier, Inserm, CHU Montpellier, Montpellier, France; 2 Faculty Of Medicine, Institute Of Clinical Medicine, Psychiatric Clinic, Vilnius University, Vilnius, Lithuania

**Keywords:** Suicide, Addiction, Substance Use Disorder

## Abstract

Suicidal behaviour is common in people with substance use disorder or behavioural addictions, and vice versa. Suicidal behaviour and addiction share many risk factors, such as increased allostatic load, and are associated with dysregulations of reward processing and impaired prefrontal cortex functioning, resulting in decision-making problems, loss of cognitive control, and impulsivity. Trait impulsivity predisposes the individual to increased sensitization to stressors or addictive stimuli. Addiction emerges when the motive for a pleasurable substance or activity transitions from positive to negative reinforcement. At this point, the stress response system is activated, and the main motivator shifts from pleasure to the escape from an aversive stimulus –withdrawal and craving. In parallel, insufferable psychological pain is the core component of the suicidal process, and a suicide attempt has been conceptualized as a way to reduce or escape it. Both states are associated with increased pain perception, stress system activation, inflammation, and anhedonia. However, while addiction generally reflects a shift from pleasure to the avoidance of negative stimuli, the pleasure is less identifiable in the suicidal process. Furthermore, not all individuals that engage in suicidal behaviours are impulsive or have an addiction, and not all individuals with addiction engage in suicidal behaviours. Yet, the understanding of the shared neurobiological component of addiction and suicidal behaviours may inform possible interventions in some individuals. Reward, pain, and stress systems are possible targets. Promising substances related to these systems that could reduce suicide risk include buprenorphine, lithium, ketamine, and psychological interventions aimed at psychological pain reduction and resilience.

**Disclosure:**

No significant relationships.

